# Projecting the Potential Distribution of *Glossina* *morsitans* (Diptera: Glossinidae) under Climate Change Using the MaxEnt Model

**DOI:** 10.3390/biology10111150

**Published:** 2021-11-08

**Authors:** Ruobing Zhou, Yuan Gao, Nan Chang, Tai Gao, Delong Ma, Chao Li, Qiyong Liu

**Affiliations:** 1 State Key Laboratory of Infectious Disease Prevention and Control, Collaborative Innovation Center for Diagnosis and Treatment of Infectious Diseases, National Institute for Communicable Disease Control and Prevention, Chinese Center for Disease Control and Prevention, Beijing 102206, China; zrb9610@126.com (R.Z.); changnan@njmu.edu.cn (N.C.); madelong97@163.com (D.M.); lichaoicdc@163.com (C.L.); 2 School of Public Health and Preventive Medicine, Monash University, Melbourne 3004, Australia; gaoyuancdc@126.com; 3 School of Public Health, Nanjing Medical University, Nanjing 210000, China; 4 Institute of Insect Sciences, College of Agriculture & Biotechnology, Zhejiang University, Hangzhou 310058, China; gaotai36@outlook.com; 5 School of Public Health, Shandong First Medical University, Jinan 250000, China

**Keywords:** tsetse fly, suitability, potentially suitable habitat

## Abstract

**Simple Summary:**

*Glossina morsitans* is a species of tsetse flies and a vector for Human African Trypanosomiasis, which is a severe parasitic infectious illness that can lead to death unless treated. At present, the *G. morsitans* are mainly found in sub-Saharan Africa. But modifications of its distribution undergoing as a result of climate change is still unknown. In order to provide scientific basis for effective monitoring and *G. morsitans* control, this study aimed to collect the distribution and to explore the potentially suitable habitat for *G. morsitans* under various scenarios. We downloaded the major data of *G. morsitans* occurrence from the Global Biodiversity Information Facility. Maxent software and R language were employed to analyze the relationship between occurrence records and climatic variables and project the potentially suitable habitat for *G. morsitans* in historical and future periods. The results showed that Isothermality contributed most to the distribution of *G. morsitans.* The predicted potentially suitable areas for *G. morsitans* under historical climate conditions include a large area of Africa near and below the equator, small equatorial regions of southern Asia, America, and Oceania. Under the future climate conditions, the potentially suitable areas would decline about −5.38 ± 1.00% as a whole under all SSPs compared with 1970–2000.

**Abstract:**

*Glossina morsitans* is a vector for Human African Trypanosomiasis (HAT), which is mainly distributed in sub-Saharan Africa at present. Our objective was to project the historical and future potentially suitable areas globally and explore the influence of climatic factors. The maximum entropy model (MaxEnt) was utilized to evaluate the contribution rates of bio-climatic factors and to project suitable habitats for *G. morsitans*. We found that Isothermality and Precipitation of Wettest Quarter contributed most to the distribution of *G. morsitans*. The predicted potentially suitable areas for *G. morsitans* under historical climate conditions would be 14.5 million km^2^, including a large area of Africa which is near and below the equator, small equatorial regions of southern Asia, America, and Oceania. Under future climate conditions, the potentially suitable areas are expected to decline by about −5.38 ± 1.00% overall, under all shared socioeconomic pathways, compared with 1970–2000. The potentially suitable habitats of *G. morsitans* may not be limited to Africa. Necessary surveillance and preventive measures should be taken in high-risk regions.

*Glossinamorsitans* (Diptera: Glossinidae), a species of tsetse flies, is 7.5 to 14 mm long and brownish-gray in color. It has scissor-like cross-wings and recognizably branched arista on the antennae. Adults have highly specialized mouthparts, which are used to bite and lap up blood [1]. Its main hosts are warthogs, oxen, buffaloes, kudus, and humans [2]. *G. morsitans* can be found in the savannah during the wet season, while, in the dry season prefer shaded wooded areas. They always remain in the lowlands [1,3]. *G. morsitans* is a vector of both Human African Trypanosomiasis (HAT) and Animal African Trypanosomiasis (AAT). HAT is a parasitic infection caused by trypanosomes, which almost invariably progresses to death unless treated [4], and which has only been found in sub-Saharan Africa until now [5]. However, less than 1000 cases of HAT were reported in 2019 due to the reinforcement of control and surveillance since 2001. It still poses a series of threats to human health, due to the severity of symptoms. Tsetse flies may disperse to new geographical areas with climate change and are notoriously difficult to treat [6].

Global warming is an indisputable fact, with an increase of about 0.89 °C (0.69–1.08 °C) over the period 1901–2012, and which is projected to increase by 0.3–4.5 °C by 2100, compared with 1986–2005 [7]. Climatic variables may influence the transmission cycle of trypanosomiasis [8]; for example, temperature drives both relative abundance of and trypanosome infections due to tsetse flies. Specifically, temperature can influence the survival and growth rate of tsetse flies [9] as well as the growth and proliferation of trypanosomes within the tsetse flies. *G. morsitans* are diurnal and are only active between the temperatures of 18–32 °C [10]. It has been observed that, in September and October, the trypanosome infection rate in *G. morsitans* is higher than in other months, due to the higher temperature [8]. Moore et al. have established a model and projected that 46–77 million additional individuals may be at risk of HAT by 2090 due to climate change [11].

*G. morsitans* is the most widespread species of the *morsitans* sub-genus, and does not live in the wettest areas (rain forest, mangrove swamp), but presents throughout much of the savanna (grassy woodland) of Africa [12]. Even though its accurate distribution is not clear enough worldwide at present, the niche model determines the relationship between species occurrence and various environmental and climatic variables, in order to create prediction distribution maps [13]. GARP, Bioclim, Domain, and MaxEnt have all been widely used to predict species distributions [14,15]; among which the maximum entropy model (MaxEnt) is effective for predicting species distributions globally. MaxEnt states that the probability distribution which best represents the current state of knowledge about a system is the one with the largest entropy, in the context of precisely stated prior data. Ahmadou et al. used species distribution models to optimize tsetse control in Senegal and found that MaxEnt predictions allow for optimizing efficiency and can be used for vector control programs [16]. Mohamed et al. predicted habitat suitability of *Culex nigripalpus* and *Cx. quinquefasciatus* in MaxEnt to provide deep insights on the competency of mosquito vector populations in transmitting the West Nile virus [17]. Ricardo et al. used MaxeEnt to project the environmental suitability of the *Aedes aegypti* under current and 2050 climatic conditions and built potential risk maps for current and future epidemiological scenarios in Brazil, in order to provide data for vector control planning [18].

Some studies have been conducted to project the distribution of *G. morsitans* in certain regions [16,19,20], and it has been reported that *G. morsitans* is endemic to 37 countries but, as of 2017, its complete distribution is still unknown, which could undergo modifications as a result of climate change [21]. Therefore, we aimed to collect the distribution and to explore potentially suitable habitats for *G. morsitans* under various scenarios. The results are expected to provide a scientific basis for effective monitoring and control of *G. morsitans* worldwide.

## 1. Materials and Methods

### 1.1. Occurrence Data

Data of *G. morsitans* occurrence were downloaded from the Global Biodiversity Information Facility (GBIF) [22]. In addition, we implemented a literature search from Pubmed and Web of Science database, in order to ensure completeness of the occurrence data. The ArcGIS software (version 10.5, ESRI Inc., Redlands, CA, USA) was used to match the approximate geographical coordinates for the available records without specific coordinates by depicting the geometric center of occurrence points in maps provided by authors or researchers. Duplicate and missing coordinate records were removed. Given that overly adjacent locations would induce overfitting, we imported all occurrence points into ArcMap and then depicted several buffers with the center as occurrence points and radius of 5 km, following which points in the same circle were deleted randomly. A total of 92 distinct locations were included in our study, 10 of which were obtained from the GBIF website and 82 of which were acquired from databases [23,24]. We used the global Moran’s I index to verify the strength of autocorrelation (Global Moran’s I index = 0.064, z-value = 59.2753, *p*-value = 0.001, permutations = 999) [25]. To meet the requirements and needs of Maxent software (version 3.4.4, http://biodiversityinformatics.amnh.org/open_source/maxent; accessed date: 15 October 2020), the distribution records were formatted as csv files.

### 1.2. Environmental Variable and Processing

The selection of environmental variables mainly depends on their restrictive effects on species distribution and the spatial correlation among variables [26]. In this study, the typical key variables—including elevation, monthly minimum temperature, monthly maximum temperature, monthly average temperature, monthly precipitation, and nineteen bioclimatic variables (bio1–bio19; see the Appendix A)—with a spatial resolution of 5 arcmin (c.10 km) and from 1970–2000 were obtained from the WorldClim-Global Climate Database (version 2.1, https://worldclim.org/; accessed on: 6 May 2021) for modeling potentially suitable areas [27].

Future environmental variables, including the nineteen bioclimatic variables, monthly minimum temperature, monthly maximum temperature, monthly average temperature, and monthly precipitation, were processed by the BCC-CSM2-MR model (a middle-resolution climate system model developed by Beijing, participated in CMIP6) under four shared socio-economic pathways (SSPs) 126, 245, 370, and 585, which were acquired from the WorldClim with the same resolution for the years 2021–2040, 2041–2060, 2061–2080, and 2081–2100. Finally, 81 data packages were downloaded.

All the environmental variables were converted into ASCII format, as required by the Maxent software.

### 1.3. Model Process

Maxent software is based on the maximum-entropy approach for modeling species niches and distributions. From a set of environmental grids and georeferenced occurrence localities, the model expresses a probability distribution where each grid cell has a predicted suitability of conditions for the species [28]. In this study, Maxent software and R language (version 3.6.0) were employed to analyze the relationship between occurrence records and climatic variables and to project the potentially suitable habitat of *G. morsitans* in historical and future periods.

### 1.4. Pre-Experiment

To reduce the random error and increase the accuracy of the prediction results, we conducted a pre-experiment. The R language was utilized to perform Pearson correlation analysis for the 24 variables, in order to reduce the risk of multicollinearity. A correlation coefficient greater than 0.8 or less than −0.8 is generally considered significant. Variables having a larger contribution rate are retained, while the others should be removed after a correlation test [29]. Nine environmental variables were reserved.

Then, the ENMeval package in R was used to test the Akaike information criterion correction (AICc), which generally provides priority to the parameters with small AIC values for simulation and is considered to be a standard to measure the goodness of model fit. Feature combination and regularization multiplier were confirmed through this process.

### 1.5. Formal Experiments

Distribution data and all environmental variables were uploaded into maxent software, and various parameters were set, including ‘Create response curves’ and ‘Random seed’. Furthermore, 75% of the distribution data was used to establish the model, and 25% were applied for testing, using bootstrap sampling, 20 replicates, 5000 maximum iterations, and 10 percentile training presence threshold rule (Table 1).

We selected the model with delta AICc equal to 0 and the corresponding parameters for *G. morsitans* according to the result of the ENMeval procedure. In Maxent software, the linear, quadratic, and product features were chosen with a 0.5 regularization multiplier. Jackknife tests were used to measure contribution rates and the importance of variables, and the ROC curve (receiver operating characteristic curve) was printed to illustrate the diagnostic ability of the binary classifier system as its discrimination threshold varied. The AUC (area under the curve) value, ranging from 0 to 1, is defined as the area under the ROC curve, which indicates the model accuracy. The model is judged to be excellent while the AUC value is between 0.9 and 1 [30]. Based on Jenks, and using the Reclassify tool of ArcMap, the probability of presence, in terms of a complementary log–log (cloglog) value, was divided into four levels: Unsuitable area (0–0.097), marginally suitable area (0.097–0.323), moderately suitable area (0.323–0.603), and highly suitable area (0.603–1).

### 1.6. Model Performance

**Figure 1:**
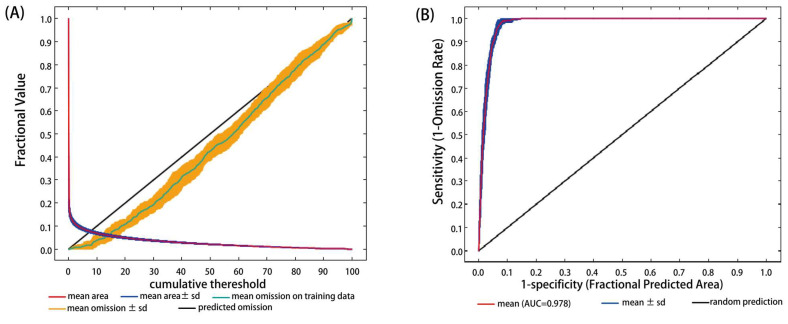
Validation charts of model performance. (**A**) Training omission rate graph; (**B**) receiver operating characteristic curve.

The training omission rate was close to the predicted omission rate under the historical situation, which means that the model was well-established (Figure 1A). The mean AUC value of ROC was 0.978 ± 0.003, indicating that the predictions were excellent in the MaxEnt model under the historical scenario (Figure 1B).

## 2. Results

### 2.1. Environmental Variable Contribution

**Figure 2:**
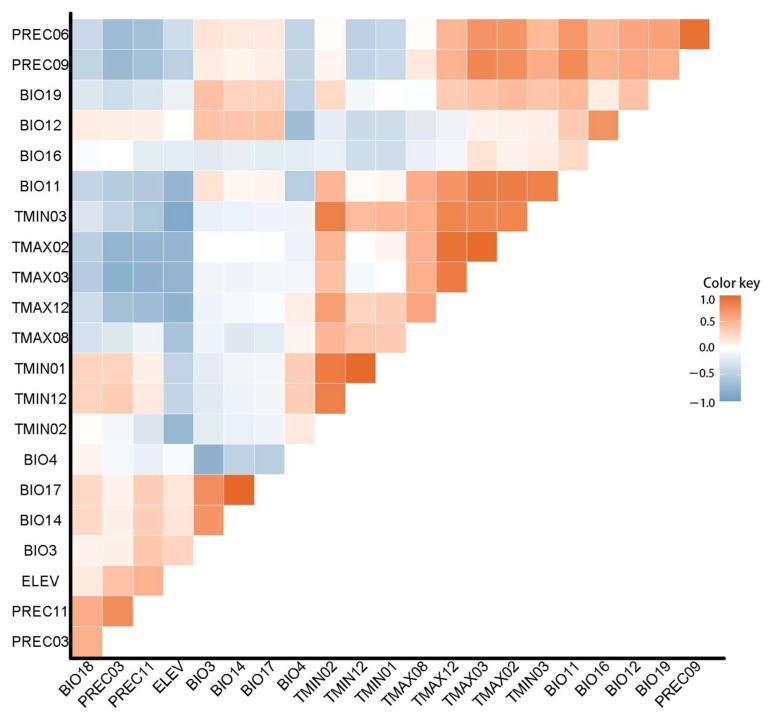
Heat map of Pearson’s correlation coefficient.

Pearson’s correlation test (Figure 2) indicated that the main factors affecting the suitability of *G. morsitans* were Isothermality, Precipitation of Wettest Quarter, Precipitation of Driest Month, Temperature Seasonality, Average Temperature of Coldest Quarter, Maximum Temperature of February, Average Precipitation of November, Average Precipitation of March, and Annual precipitation, with contributions of 26.4%, 22.4%, 6.8%, 4.9%, 4.6%, 4.3%, 3.2%, 2.7%, and 2.4%, respectively (Table 2). Isothermality quantifies how drastically the day-to-night temperatures oscillate relative to the summer-to-winter (annual) oscillations (an isothermal value of 100 indicates the diurnal temperature range is equivalent to the annual temperature range, while anything less than 100 indicates a smaller level of temperature variability within an average month relative to the year). The total contribution rate of these nine factors was 77.7% in the pre-experiment.

**Figure 3:**
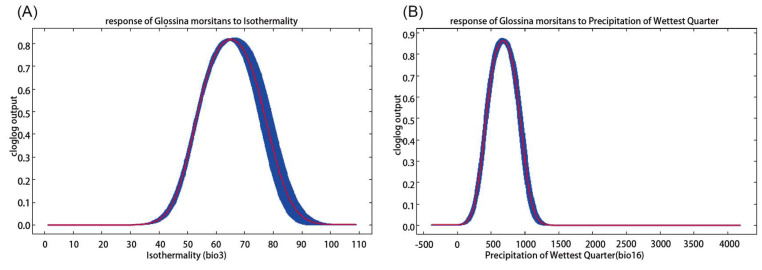
Response curves for dominant climatic factors.

Given the highest contribution rates of bio3 and bio16, we conducted a single analysis to illustrate their single factor influence. As shown in Figure 3A, with the increase in Isothermality the probability of *G. morsitans* presence increased first and decreased later. When Isothermality lay within the 55–75% interval, the probability was higher than 0.6, corresponding to a highly suitable area; while the probability of presence reached its highest value (about 0.84) when Isothermality was around 65%.

The Precipitation of Wettest Quarter for highly suitable habitats ranged from 490 mm to 900 mm with the peak (around 0.88) of 600 mm (Figure 3B).

### 2.2. Potential Distribution of G. morsitans under Historical Conditions

**Figure 4:**
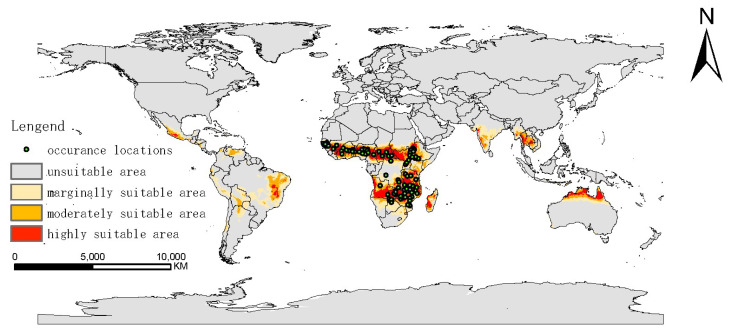
Occurrence locations of *Glossina morsitans* and potentially suitable area under historical situation. (probability: unsuitable area 0–0.097; marginally suitable are 0.097–0.323; moderately suitable area 0.323–0.603; highly suitable area 0.603–1).

The occurrence points of *G. morsitans* are mainly situated in low-latitude areas of Africa. The predicted potentially suitable area for *G. morsitans* under historical climate conditions would be 14.5 million km^2^, including a large part of Africa near and below the equator, as well as small equatorial regions of southern Asia, America, and Oceania. In all suitable areas, the proportions of highly suitable area, moderately suitable area, and marginally suitable area were 25.94%, 29.67%, and 44.39%, respectively. In detail, highly, moderately, and marginally suitable areas were situated in 33, 46, 53 countries, respectively. With highly and moderately suitable areas mainly distributed in Western, Eastern, and Southern Africa (mainly including the Central African Republic, Chad, Zambia, Tanzania, Senegal, Nigeria, South Sudan, Mozambique, Mali, Malawi, Madagascar, Cote d ‘Ivoire, Cameroon, Zimbabwe, Ghana, Guinea, Gambia, Angola, and Ethiopia), southern Asia (including the west of India, Thailand, Cambodia, Myanmar, and Laos), the eastern part of Brazil, the southern part of Mexico, and the very northern part of Australia. Marginally suitable areas were not only distributed around moderately and highly suitable areas but also in the middle and north of South America (including Chile, Peru, Ecuador, and Bolivia), North America (including Nicaragua, Honduras, Haiti, Cuba, and Dominica) and Vietnam, South Asia (Figure 4).

### 2.3. Potential Distribution of G. morsitans under Future Conditions

**Figure 5:**
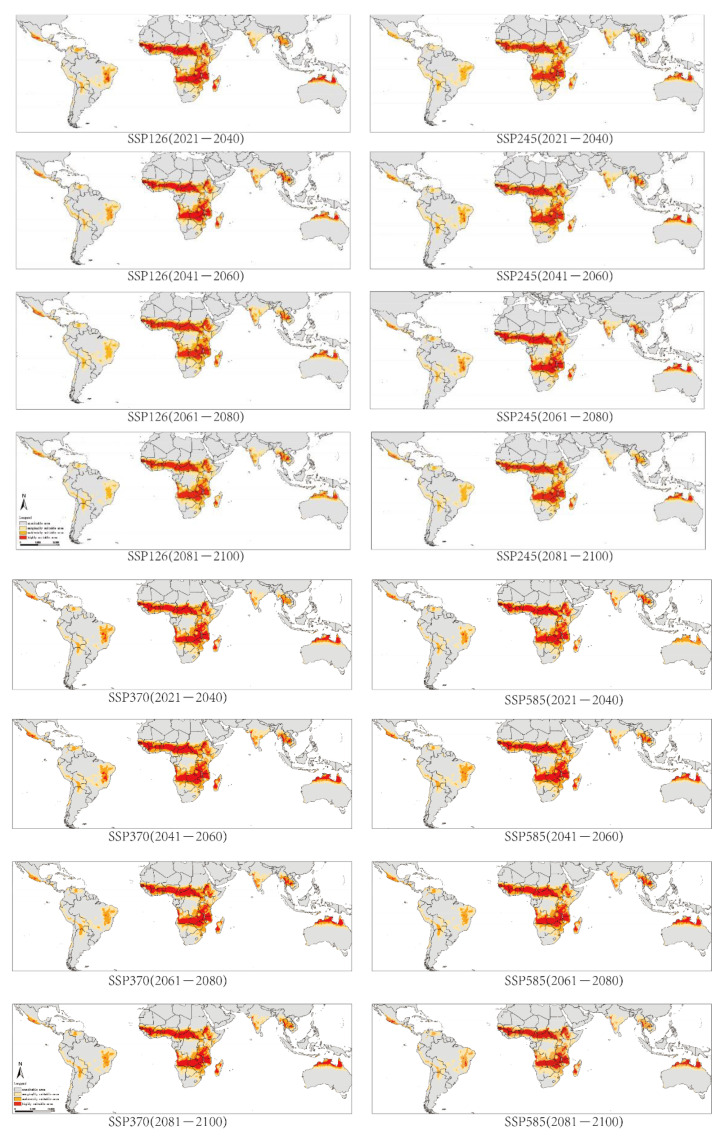
Potentially suitable area for *Glossina morsitans* under four scenarios from 2021–2100.

The projected distribution range of *G. morsitans* would not change a lot, but the amount of area would change to some extent. For example, under the SSP126 scenario, the highly suitable area in Brazil would decrease from 94.22 thousand km^2^ in 2021–2040 to 2.25 thousand km^2^ in 2081–2100. In contrast, the highly suitable area in Thailand would increase from 77.19 thousand km^2^ to 111.88 thousand km^2^ over the same period (Figure 5).

### 2.4. The Trend of Prediction Area of G. morsitans

#### 2.4.1. The Overall Trend of Potentially Suitable Areas

Under future climate conditions, the potentially suitable total areas would decline by about −5.38 ± 1.00% overall SSPs, compared with 1970–2000. For the SSP126 scenario, the area ranges from 15.34 million km^2^ in 2061–2080 to 13.8 million km^2^ in 2081–2100. For the SSP245 scenario, the area would peak at 15.42 million km^2^ in 2041–2060, then decrease to 13.44 million km^2^ in 2081––2100.

**Figure 6:**
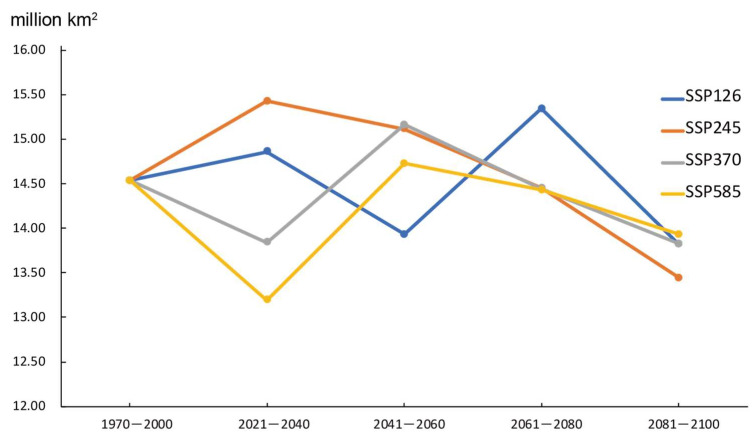
Variation trend of the amount of potentially suitable area.

The same variation tendency would be seen for SSP370 and SSP585 scenarios. They would decrease first and peak in 2041–2060 (Figure 6).

#### 2.4.2. Secular Trend of Suitable Area for Different Periods

While the amount of highly suitable area would decrease from roughly 3.77 million km^2^ to 3.38 million km^2^, on average, within 100 years under all four scenarios in the whole. For the SSP126 scenario, the amount of highly suitable area would decline to approximately 3.24 million km^2^ in the 2041–2060 period, then increase to 3.68 million km^2^ in 2061–2080. The amount of area would peak in 2041–2060 under SSP245 or SSP370, while the peak period for SSP585 would be 2061–2080.

**Figure 7:**
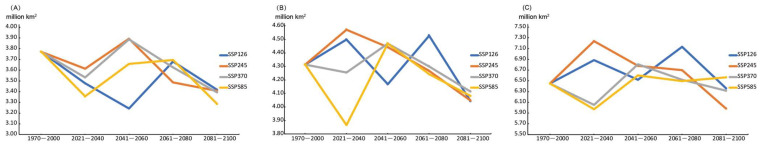
Variation trend of the amount of potentially suitable area under four scenarios. ((**A**) highly suitable area; (**B**) moderately suitable area; (**C**) marginally suitable area).

The amount of moderately suitable area would also decline in general, from 4.21 million km^2^ in 1970–2000 to 4.07 million km^2^ on average in 2081–2100. For marginally suitable habitats, the amount of marginally suitable area would decline a little under almost all SSPs, except for the SSP585 scenario. Under this scenario, the amount would decrease from 6.45 million km^2^ to 5.79 million km^2^, then increase to 6.57 million km^2^ in 2100 (Figure 7).

## 3. Discussion

### 3.1. Discussion of Major Environmental Factors and Prediction Results

It has been known for a long time that the general distribution of *G.morsitans* is determined by climate and influenced by altitude, vegetation, human activities, and the presence of host animals [31]. It has been recognized that climate is an important factor in determining the distribution of *G. morsitans* [32]. In our study, we found that Isothermality contributed most to the distribution of *G. morsitans*, and the appropriate interval was 40–100%. The possible reason for this is that appropriate Isothermality is beneficial to the survival and reproduction of *G. morsitans* [32]. Isothermality has a strong influence on maintaining population survival, and could even compensate for the negative effects of other factors, such as inappropriate precipitation, affecting pupae [33]. Stable temperatures (23–25 °C) also provide an optimal environment for pupal fat accumulation and development [34], which is crucial for their adult period. Furthermore, the distribution of *G. morsitans* might be limited by the exhaustion of fat reserves during development under extreme climatic conditions [32]. We also found the Precipitation of Wettest Quarter factor to strongly contribute to their distribution. Precipitation is another crucial factor determining the distribution of *G. morsitans*. In our study, comparatively low Precipitation of Wettest Quarter (200–1500 mm) was suitable for the survival of *G. morsitans*. A possible reason for this is that, in years with exceptionally heavy rainfall, the inundation of downstream breeding grounds leads to dramatic reductions in the population [32]. Additionally, it is thought that high rainfall may cause local flooding, which may wash out pupae that are buried in loose soil [35]. Thus, we conjecture that the precipitation of the wettest quarter could limit the distribution to a large extent.

### 3.2. Discussion of Potential Distribution of G.morsitans under Historical Conditions

Under the historical situation, we predicted potentially suitable habitats of *G. morsitans*, including mainly low-latitude areas of Arica, central and southern South America, southern North America, southern Asia, and northern Australia. Our results for Africa correspond with the predictions for the potential range of *G. morsitans* in Africa made by the Food and Agriculture Organization of the United Nations (FAO) in 1999 and 2017 [36,37,38], with slight differences which may be due to the distinct sampling locations. Furthermore, the limit of distribution is closely correlated with the tropical savanna climate (summer rain), which follows the 508 mm annual isohyet. This result is consistent with an ecological survey carried out by Langridge [32], which indicated that districts covered by tropical savanna climate were relatively vulnerable to *G. morsitans*. These districts also comprised the HAT epidemic area [39]. Thus, we suggest that people who live in these areas should strengthen the public awareness, education, diagnosis, and treatment for *G. morsitans* and HAT.

### 3.3. Discussion of Potential Distribution of G.morsitans under Future Conditions

In 2021–2100, the amount of highly suitable area presented an overall trend of increasing first, to a peak, and then decreasing from 2041–2060 under all scenarios—except for SSP126, which would reach its peak in the 2061–2080 period. This may be as the optimum environment for *G. morsitans* comes earlier with climate warming, under the SSP245, SSP370, and SSP585 scenarios, which means that, with global warming becoming more serious, we expect that there will be more suitable habitat areas for *G. morsitans* first; however, global warming will eventually become a negative factor for *G. morsitans*. Meanwhile, under the SSP126 scenario, the optimum time comes later (2061–2080), as it becomes warmer more slowly.

### 3.4. Advantages and Disadvantages

The advantage of our study was that we provided scientific information for the prediction of *G. morsitans* distribution from a global aspect. In addition, the ENMeval package in R language was utilized to optimize the RM value and feature combination; this tuning exercise can result in a model with a balanced goodness-of-fit.

However, there were some limitations. First, the bioclimatic data obtained from WorldClim were only uploaded until 2000, instead of the latest period. Second, other variables which may affect the distribution of *G. morsitans* were not included in our model prediction based on data restrictions. For example, vegetation is a vital factor for providing shade and maintaining a suitable microclimate for *G. morsitans*, as well as habitat for their hosts.

## 4. Conclusions

Our study provided scientific information for the prediction of *G. morsitans* distribution from a global standpoint. We found that Isothermality and Precipitation of Wettest Quarter contributed most to the distribution of *G. morsitans*. The predicted potentially suitable areas for *G. morsitans* under historical climate conditions would be 14.5 million km^2^, including a large area of Africa near and below the equator, as well as small equatorial regions of southern Asia, America, and Oceania. Under future climate conditions, the potentially suitable areas are expected to decline by about −5.38 ± 1.00% overall under all SSPs, compared with 1970–2000. In the future, more environmental, social-economic, and anthropogenic variables should be included to model the projected distribution of *G. morsitans* in high-risk regions. At the same time, predicted suitable areas for *G. morsitans* without any records should strengthen surveillance of their associated and alarm systems.

## Figures and Tables

**Table 1:** Maxent parameter settings.

Option	Parameter	
	Default Value	Setting Value
Randomly selected test set percentage	0	25
Regularization multiplier	1	0.5
Replicated run type	Crossvalidate	Bootstrap
Number of iterations repeated	1	20
Maximum number of repetitions	500	5000
Apply threshold rules	None	10 percentile training presence
Features	Auto (hinge, product, quadratic, linear)	Liner, quadratic, product

**Table 2:** Nine screened climatic factors and their contribution rates.

Abbreviation	Definition	Contribution Rate (%)
bio3 *	Isothermality (Bio2/Bio7 × 100) (%) ^#^	26.4
bio16 *	Precipitation of Wettest Quarter (mm)	22.4
bio14	Precipitation of Driest Month (mm)	6.8
bio4	Temperature Seasonality (standard deviation ∗ 100)	4.9
bio11	Average Temperature of Coldest Quarter (°C)	4.6
tmax02	Maximum Temperature of February (°C)	4.3
prec11	Average Precipitation of November (mm)	3.2
prec03	Average Precipitation of March (mm)	2.7
bio12	Annual Precipitation (mm)	2.4

^#^ Bio2 = Mean Diurnal Range [Mean of monthly (max temperature—min temperature)]. ^#^ Bio7 = Temperature Annual Range (Max Temperature of Warmest Month—Min Temperature of Coldest Month). * Variables for single analysis.

## Data Availability

The bioclimatic data used in this study can be downloaded at https://www.worldclim.org/; accessed on 6 May 2021. The occurrence records used in this study can be downloaded at https://www.gbif.org; accessed on 1 April 2021.

## Appendix A

**Table A1:** Definitions of the Nineteen Bioclimatic Variables.

BIO1 = Annual Mean Temperature
BIO2 = Mean Diurnal Range [Mean of monthly (max temp − min temp)]
BIO3 = Isothermality [(BIO2/BIO7) × 100]
BIO4 = Temperature Seasonality (standard deviation ×100)
BIO5 = Max Temperature of Warmest Month
BIO6 = Min Temperature of Coldest Month
BIO7 = Temperature Annual Range (BIO5 − BIO6)
BIO8 = Mean Temperature of Wettest Quarter
BIO9 = Mean Temperature of Driest Quarter
BIO10 = Mean Temperature of Warmest Quarter
BIO11 = Mean Temperature of Coldest Quarter
BIO12 = Annual Precipitation
BIO13 = Precipitation of Wettest Month
BIO14 = Precipitation of Driest Month
BIO15 = Precipitation Seasonality (Coefficient of Variation)
BIO16 = Precipitation of Wettest Quarter
BIO17 = Precipitation of Driest Quarter
BIO18 = Precipitation of Warmest Quarter
BIO19 = Precipitation of Coldest Quarter
